# Feasibility of an outpatient‐based pulmonary rehabilitation program for lung cancer patients during radiation therapy

**DOI:** 10.1111/1759-7714.14061

**Published:** 2021-06-30

**Authors:** Hye Joon Ahn, Jae Yong Jeon, Su Ssan Kim, Si Yeol Song, Won Kim, Sei Won Lee, A Hyun Kim, Seung Hak Lee

**Affiliations:** ^1^ Department of Rehabilitation Medicine, Asan Medical Center University of Ulsan College of Medicine Seoul South Korea; ^2^ Department of Radiation Oncology, Asan Medical Center University of Ulsan College of Medicine Seoul South Korea; ^3^ Department of Pulmonary and Critical Care Medicine, Asan Medical Center University of Ulsan College of Medicine Seoul South Korea

**Keywords:** lung cancer, pulmonary rehabilitation, radiation therapy

## Abstract

**Purpose:**

Data are lacking regarding pulmonary rehabilitation (PR) programs in patients with lung cancer receiving radiation therapy. This study aimed to confirm the feasibility of an outpatient‐based PR program in lung cancer patients during radiation therapy.

**Methods:**

A retrospective chart review was performed of 40 patients with lung cancer who had undergone radiation therapy between July and December 2019. The patients received an outpatient‐based PR program for a total of eight sessions two times weekly comprising 60 min per session. Feasibility was assessed based on the completion rate, adverse events, and satisfaction with the PR program. Functional evaluations using 6‐min walk and grip strength tests were conducted before and after PR. Patient quality of life was assessed by the EORTC QLQ‐C30 questionnaire before and after PR.

**Results:**

The completion rate for the PR program was 72.5% among the 40 patients. No adverse events related to PR were reported. The overall satisfaction was 5.7 ± 1.1 on a seven‐point Likert scale in all participants. The mean 6‐min walk test distance increased significantly from 419.1 to 446.2 m. The improvement in grip strength in the dominant hand after PR was not significant. The social functioning score in the EORTC QLQ‐C30 improved significantly.

**Conclusion:**

The results of this study showed the feasibility without serious adverse effects of a 4‐week outpatient‐based PR program for lung cancer patients undergoing outpatient‐based radiation therapy. This program might improve patient physical function and quality of life.

## Graphical abstract

The results of this study show the feasibility without serious adverse effects of a 4‐week outpatient‐based pulmonary rehabilitation program for lung cancer patients during outpatient‐based radiation therapy.

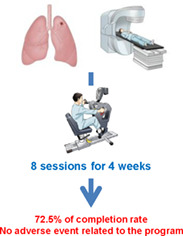

**Abbreviations**
6MWT: 6‐minute walk test
CCRT: concurrent chemoradiotherapy
COPD: chronic obstructive pulmonary disease
ECOG PS: Eastern Cooperative Oncology Group performance status
EORTC QLQ‐C30: European Organization for Research and Treatment of Cancer Quality of Life Questionnaire Core 30
FEV1: forced expiratory volume in 1 s
FVC: forced vital capacity
NSCLC: non‐small cell lung cancer
PR: pulmonary rehabilitation
RILI: radiation‐induced lung injury
RIPF: radiation‐induced pulmonary fibrosis
RPE: rating of perceived exertion

## INTRODUCTION

Pulmonary rehabilitation (PR) is a multidisciplinary and comprehensive program for people with chronic respiratory diseases.^1^ In chronic obstructive pulmonary disease (COPD), PR reportedly improves respiratory distress, exercise performance, quality of life, and cost‐effectiveness.^2^, ^3^ Although not as evidenced as COPD, there are some reports of the effectiveness of PR programs in patients with interstitial lung disease,^4^ bronchiectasis,^5^ asthma,^6^ and pulmonary artery hypertension.^7^ The 2019 Cochrane Review concluded that exercise training following lung resection for non‐small‐cell lung cancer (NSCLC) improves exercise capacity, health‐related quality of life, and the symptom of dyspnea.^8^ A review of studies on preoperative exercise training for patients with NSCLC concluded that exercise training before lung surgery improved exercise capacity and forced vital capacity (FVC).^9^ However, there are few reports of PR programs in lung cancer patients treated with chemotherapy or radiation therapy.^10^, ^11^, ^12^, ^13^, ^14^, ^15^

While radiation therapy is an important treatment modality for lung cancer, it can cause radiation‐induced lung injury (RILI), including pneumonitis or radiation‐induced pulmonary fibrosis (RIPF), and pneumonia, and pulmonary function declines after radiation therapy.^16^, ^17^ Although there are not many studies regarding PR programs in patients with lung cancer receiving radiation therapy, some studies have shown that PR programs may be beneficial to lung cancer patients undergoing radiation therapy. One retrospective matched case‐control study regarding simultaneous PR during thoracic radiation therapy in patients with lung cancer or esophageal cancer showed that a simultaneous PR program improved pulmonary function and exercise capacity.^13^ Another preliminary study demonstrated that PR programs improved exercise tolerance and quality of life, as measured by the 6‐min walking distance test and the European Organization for Research and Treatment of Cancer Quality of Life Questionnaire Core 30 (EORTC QLQ‐C30), respectively, among inpatients receiving concurrent chemoradiotherapy (CCRT) who performed incremental symptom‐limited exercise using cycling and treadmill.^14^ The other study on perioperative PR for NSCLC patients with CCRT before pulmonary resection included relaxation, respiratory training, cough training, lower‐extremity exercise, and training in activities of daily living, and reported that 10 weeks of PR significantly increases pulmonary function, particularly in smokers and those with respiratory impairment.^15^ These studies showed the benefits of PR programs in lung cancer patients receiving radiation therapy, including improved exercise tolerance, pulmonary function, and quality of life.

In our center, the departments related to radiation therapy have constant concerns regarding how to care for patients experiencing complications of thoracic radiation therapy. Even though there is not sufficient evidence regarding the effects of PR in lung cancer patients receiving radiation therapy, some reports have shown that PR may improve pulmonary and physical function and quality of life. Therefore, our center developed an outpatient‐based PR program for lung cancer patients undergoing outpatient‐based radiation therapy. We expected that this outpatient‐based program would positively affect physical function and quality of life in these patients. This study aimed to confirm the feasibility of this program.

## METHODS

### Participants

This retrospective feasibility study reviewed the medical records of patients diagnosed with lung cancer from an outpatient clinic of the Rehabilitation Medicine Department in our hospital. According to pathology and stage, pulmonologists and radiation oncologists determined the radiation therapy plan, including definitive, palliative, salvage, preoperation, and postoperation radiation therapy. Before starting radiation therapy, the patients were referred to the rehabilitation department for the PR program. Eastern Cooperative Oncology Group performance status (ECOG PS) was evaluated by one physician at the first visit. All patients were over 19 years of age and received outpatient‐based PR between July and December 2019.

### Outpatient‐based PR program and assessments

This outpatient‐based PR rehabilitation program commenced from July 2019 for lung cancer patients who underwent outpatient‐based radiation therapy. The program was conducted simultaneously during the radiation therapy period. Functional evaluation was conducted using a 6‐min walk test (6MWT) and a grip strength test before and after PR. After functional evaluations, the 4‐week outpatient‐based PR program was started in conjunction with outpatient‐based radiation therapy. The outpatient‐based PR program lasted for four consecutive weeks, with two sessions per week. Each 60‐min exercise session was conducted under the supervision and guidance of a physical therapist. Most previous studies of PR in lung cancer patients administering aerobic and/or resistance exercise with or without inspiratory muscle training^8^, ^9^ referred to PR for COPD, which includes physical activity considered to be aerobically demanding. Each session of our PR program was performed in the following order: warm‐up (10 min), strengthening exercise (20 min), aerobic exercise (20 min), and cool‐down (10 min). The strengthening exercises included squats, bridge exercises, bird‐dog exercises, and leg lowering drills at an intensity of 13–15 points of the rating of perceived exertion (RPE). The aerobic exercise used a fixed cycle at an intensity of 65–85% of the maximum heart rate (based on age), with a 13–15 point intensity according to the Borg scale. During each session, a physical therapist monitored the patients for side effects including fatigue, breathing difficulty, and muscle pain. The completion rate was assessed based on completion of the eight sessions of the PR program. Additionally, the adherence rate was assessed as the ratio of the number of sessions that patients actually participated in, compared to the total number of sessions conducted. After all programs were completed, the usability of the PR program was assessed using a self‐reported questionnaire with a seven‐point Likert scale on six items (overall satisfaction, safety, helpfulness to radiation therapy, willingness to participate again even if the patient does not come to receive radiation therapy on daily basis, willingness to participate again if the patient returns to the initial stage of radiation therapy, and physical function improvement). The EORTC QLQ‐C30 questionnaire was administered before and after the PR program. All patients visited the outpatient clinic of the rehabilitation department three times: before the program, at the initial stage of the program, and before the cessation of the program. At the first visit, the physician educated the patients about complications of radiation therapy including pneumonitis and RIPF and the necessity of the PR program, and they were encouraged to participate in the program. At the second and final visits, the results of initial and follow‐up functional evaluation, respectively, were explained to the patients, and the physician encouraged them to maintain physical activity after cessation of the PR program.

### Statistical analysis

Statistical analysis was performed using IBM SPSS Statistics for Windows, version 25.0 (IBM Corp.) and representative values were expressed as means ± standard deviation and percentages, respectively. Paired *t*‐tests were used to compare changes in the variables from baseline to the completion of the PR program. *p* values <0.05 were considered statistically significant.

## RESULTS

Twenty‐nine of the 40 patients who started the PR program completed the program (21 male, eight female; median age 65.8 years). Nineteen patients were smokers and 26 were diagnosed with NSCLC. Among NSCLC patients, 53.8% and 30.8% had stage IIIA and IIIB disease, respectively. The baseline ECOG PS scores were PS1 (82.8% of patients) and PS 2 (17.2%). Eleven patients underwent lung resection surgery. Patients who underwent surgery received radiation therapy and participated in the PR program after surgery. The initial FVC predicted value (%) and forced expiratory volume in 1 s (FEV1)/FVC (%) were 76.11% and 0.72, respectively (Table 1).

**TABLE 1:** Baseline participant characteristics and functional outcomes before and after the pulmonary rehabilitation program

Variables	Values	Variables	Values
Age (years), median (IQR)	65.8 ± 8.4	Pathology (*n*, %)	
Male (*n*, %)	21 (72.4)	NSCLC	26 (89.7)
Smoking (*n*, %)		SCLC	3 (10.3)
Never	10 (34.5)	Stage (*n*, %), NSCLC	
Ever	19 (65.5)	I	1 (3.8)
ECOG PS (*n*, %)		II	3 (11.5)
0	0 (0.0)	IIIA	14 (53.8)
1	24 (82.8)	IIIB	8 (30.8)
2	5 (17.2)	IV	0 (0.0)
Underlying lung disease (*n*, %)		Stage (*n*, %), SCLC	
COPD	8 (27.6)	Limited disease	2 (66.7)
Asthma	0 (0.0)	Extended disease	1 (33.3)
ILD	0 (0.0)	Pulmonary function test	
Purpose of RT (*n*, %)		FVC (L)	3.045 ± 0.64
Definitive	17 (58.6)	FVC (%)	77.64 ± 11.95
Palliative	0 (0.0)	FEV1 (L)	2.16 ± 0.40
Salvage	1 (3.4)	FEV1 (%)	76.11 ± 12.24
Postoperation	11 (37.9)	FEV1/FVC	0.72 ± 0.08
Others	0 (0.0)	DLCO (mL/mmHg/min)	14.58 ± 4.67
RT duration, days	27.5 ± 4.1	DLCO (%)	72.92 ± 19.08
RT fraction, cGY	5789.7 ± 607.3		

*Abbreviations*: COPD, chronic obstructive pulmonary disease; DLCO, diffusing capacity for carbon monoxide; ECOG PS, Eastern Cooperative Oncology Group performance status; FEV1, forced expiratory volume in 1 s; FVC, forced vital capacity; GY, gray; ILD, interstitial lung disease; NSCLC, non‐small cell lung cancer; RT, radiation therapy; SCLC, small cell lung cancer.

The completion rate was 72.5% (29 out of 40 patients). The adherence rate was 81.25% (260 out of total 400 sessions). No adverse events related to PR were observed, including cardiac or pulmonary instability, dizziness, extreme fatigue, or musculoskeletal problems. Self‐reported questionnaires were obtained from 20 patients who completed the program, with scores of 5 or higher on all items (Table 2). The overall satisfaction was 5.7 ± 1.1 and the self‐reported helpfulness of the PR program for undergoing radiation therapy had the highest score (6.1 ± 0.9), whereas the willingness to participate in a PR program even if the patient does not receive the outpatient‐based radiation therapy on daily basis had the lowest score (5.5 ± 1.3). The other items scored as follows: “How anxious about safety accidents during outpatient‐based PR program” (5.9 ± 1.3), “Are you willing to participate in the outpatient‐based PR program again if you return to the initial stage of radiation therapy?” (5.7 ± 1.4), and “How do you expect the outpatient‐based PR program to help you improve your physical performance in the future?” (5.7 ± 1.1). The mean 6MWT distance increased significantly from 419.1 to 446.2 m. The grip strength increased significantly in the nondominant hand after the PR program; however, the improvement was not statistically significant in the dominant hand. Significant improvement in social functioning on the EORTC QLQ‐C30 was observed; however, the other scales, including global health status, functional scales (physical functioning, role functioning, emotional functioning, cognitive functioning), symptom scales (fatigue, nausea and vomiting, pain, dyspnea, insomnia, appetite loss, constipation, diarrhea, financial difficulties) did not show significant improvement (Table 3).

**TABLE 2:** Usability assessment results of the PR program by a self‐reported questionnaire using a seven‐point Likert scale

Self‐reported questionnaire items	Results
Overall satisfaction with the PR program	5.7 ± 1.1
Self‐reported helpfulness of the PR program for undergoing radiation therapy	6.1 ± 0.9
Anxiousness about safety accidents during the outpatient‐based PR program	5.9 ± 1.3
Willingness to participate again if the patient returns to the initial stage of radiation therapy	5.7 ± 1.4
Expectation of helpfulness of the PR program to improve physical performance in the future	5.7 ± 1.1
Willingness to participate in the PR program even if the patient does not receive outpatient‐based radiation therapy on daily basis	5.5 ± 1.3

*Abbreviation*: PR, pulmonary rehabilitation.

**TABLE 3:** Results of functional outcomes and EORTC QLQ‐C30 before and after the PR program in lung cancer patients receiving radiation therapy

Variables	Values		
	Pre‐rehabilitation	Post‐rehabilitation	*p* value
6MWT (m)	419.1 ± 103.0	446.2 ± 117.3	<0.001*
Grip strength, dominant (kg)	30.3 ± 8.8	30.8 ± 8.6	0.543
Grip strength, nondominant (kg)	27.7 ± 8.1	29.3 ± 8.8	0.021*
EORTC QLQ‐C30			
Global health status/QoL			
Global health status/QoL	57.2 ± 19.5	60.1 ± 23.5	0.447
Functional scales			
Physical functioning	77.7 ± 18.3	80.7 ± 13.1	0.190
Role functioning	75.9 ± 23.4	79.3 ± 17.9	0.440
Emotional functioning	79.6 ± 18.8	80.7 ± 15.8	0.647
Cognitive functioning	79.9 ± 16.6	80.5 ± 15.8	0.840
Social functioning	69.5 ± 22.8	81.0 ± 21.3	0.041*
Symptom scales			
Fatigue	27.6 ± 14.2	29.1 ± 14.1	0.542
Nausea and vomiting	9.2 ± 14.2	11.5 ± 14.6	0.532
Pain	19.5 ± 14.6	20.7 ± 24.6	0.775
Dyspnea	25.3 ± 22.6	27.6 ± 26.4	0.755
Insomnia	19.5 ± 24.0	27.6 ± 21.6	0.151
Appetite loss	26.4 ± 23.8	26.4 ± 23.8	–**
Constipation	21.8 ± 23.6	25.3 ± 30.0	0.508
Diarrhea	9.2 ± 14.9	10.3 ± 19.8	0.794
Financial difficulties	23.0 ± 26.4	20.7 ± 22.2	0.613

*Abbreviations*: 6MWT, 6‐min walking test; EORTC QLQ‐C30, the European Organization for Research and Treatment of Cancer Quality of Life Questionnaire Core 30; PR, pulmonary rehabilitation.

* Paired *t*‐test.

** Paired *t*‐test result could not be derived because there was no significant difference between the groups (before and after the PR program).

## DISCUSSION

Our results showed that an outpatient‐based PR program was feasible and might improve physical function in lung cancer patients receiving radiation therapy. No serious adverse events were noted and the overall satisfaction was high. Most previous studies on PR in lung cancer patients were conducted in preoperative or postoperative conditions.^18^, ^19^, ^20^ Most previous studies regarding PR programs for lung cancer patients receiving radiation therapy included only inpatients.^14^, ^15^ This study is the first to evaluate the feasibility and safety of an outpatient‐based PR program for lung cancer patients undergoing outpatient‐based radiation therapy.

Since our PR program included only outpatients, the completion rate was the most important outcome. Twenty‐nine of 40 patients (72.5%) completed all 4 weeks of the outpatient‐based PR program, indicating the program feasibility. Previous studies of outpatient‐based PR programs in COPD patients reported completion rates varying from approximately 40% to 66%.^21^, ^22^, ^23^ The factors contributing to poor adherence in previous studies included transportation problems, lack of motivation, work‐related reasons, hospitalization, aggravation of underlying disease, and very low exercise capacity.^21^, ^24^, ^25^ One feasibility study of an outpatient‐based PR program for patients receiving chemotherapy reported a completion rate of 75%, in which accessibility and unwillingness were the reasons for refusing the PR program.^12^ We observed a relatively higher completion rate than those in previous studies. The patients who participated in our program were directed to visit the hospital daily for outpatient‐based radiation therapy and the PR program was conducted on some of the days of the patients' hospital visits for radiation therapy. We believe that the simultaneous manner of our PR program during radiation therapy may have contributed to the high completion rate. Moreover, most patients living far from the hospital rented temporary residences near the hospital, therefore accessibility was not a barrier for patients receiving daily outpatient‐based radiation therapy. The reasons for patients refusing our program were unwillingness and poor medical conditions. Compared to outpatient‐based PR programs in patients with other medical conditions, including COPD and lung cancer with chemotherapy (pre‐operation or post‐operation), this simultaneous PR program during radiation therapy in lung cancer patients was more advantageous for patient participation because radiation therapy was administered daily.

The median 6MWT distance increased significantly from 419.1 to 446.2 m. The grip strength of the nondominant hand improved significantly after the PR program. While the grip strength also improved on the dominant hand after the PR program, the difference was not statistically significant. Because our study had no control group, it is limited in showing the functional benefits of the PR program. However, one retrospective study using a control group also reported the improvement of 6MWT distance after a simultaneous PR program during thoracic radiation, which is in line with our result.^13^ Additional prospective randomized controlled trials are needed to evaluate the functional benefits of PR programs. The results of the self‐reported questionnaire with a seven‐point Likert scale showed high participant satisfaction and modest levels of safety. The satisfaction and safety reported by patients support the feasibility of this outpatient‐based PR program.

Regarding the patient quality of life, significant improvement was observed in the social functioning scale of the EORTC QLQ‐C30, but not for the global health status, symptom scales, and functional scales except social functioning before and after the PR program. One study assessing patient quality of life after thoracic radiation therapy showed worsening for every score for global health status and symptom scales and for some of the functional scales (physical functioning, role functioning, cognitive functioning, and social functioning) in the EORTC QLQ‐C30 after 6 weeks of radiation therapy.^26^ In our study, every scale in the EORTC QLQ‐C30 was consistent before and after the outpatient‐based PR program, except for the improvement in social functioning. These results indicate that the outpatient‐based PR program can prevent the deterioration of quality of life following thoracic radiation therapy. Regarding long‐term lung cancer survivors, over one‐third of patients experience significantly impaired quality of life, with worsened symptoms including fatigue, pain, dyspnea, depressed appetite, and coughing over time.^27^ While the results of our study show the potential for an outpatient‐based PR program to prevent a short‐term decline in patient quality of life, further study is warranted to evaluate the long‐term effects of the PR program to prevent the deterioration of patient quality of life.

### Study limitations

First, the main limitation of this study is the lack of a control group. Because this study was a single‐arm study limited to patients undergoing PR, it is difficult to give sufficient validity to the results, including the improvement in physical function and no deterioration of quality of life after PR. Second, we could not compare pulmonary function before and after the PR program, thus we were unable to evaluate the improvement in pulmonary function after the PR program. Further prospective studies with randomized controlled designs are needed to evaluate changes in pulmonary and physical function and quality of life after completing a PR program. Additionally, in this study radiation therapy and pulmonary rehabilitation were performed in the same building, therefore high compliance to the PR program, which is the main result of this study, may be difficult to generalize and apply to other regions and medical environments.

### Conclusion

The results of this study show that a 4‐week outpatient‐based PR program for lung cancer patients undergoing outpatient‐based radiation therapy is feasible, without serious adverse effects. Furthermore, this program might improve patient physical function and quality of life.

## CONFLICT OF INTEREST STATEMENT

None.

## FINANCIAL SUPPORT

None.
